# Maintenance of intestinal homeostasis by mucosal barriers

**DOI:** 10.1186/s41232-018-0063-z

**Published:** 2018-04-02

**Authors:** Ryu Okumura, Kiyoshi Takeda

**Affiliations:** 1 Department of Microbiology and Immunology, Graduate School of Medicine, Osaka University, Osaka, 565-0871 Japan; 2 WPI Immunology Frontier Research Center, Osaka University, Osaka, 565-0871 Japan; 3 Core Research for Evolutional Science and Technology, Japan Agency for Medical Research and Development, Tokyo, 100-0004 Japan

**Keywords:** Mucosal barrier, Gut microbiota, Intestinal epithelial cells, Inflammatory bowel disease

## Abstract

**Background:**

The intestine is inhabited by a tremendous number of microorganisms, which provide many benefits to nutrition, metabolism and immunity. Mucosal barriers by intestinal epithelial cells make it possible to maintain the symbiotic relationship between the gut microbiota and the host by separating them. Recent evidence indicates that mucosal barrier dysfunction contributes to the development of inflammatory bowel disease (IBD). In this review, we focus on the mechanisms by which mucosal barriers maintain gut homeostasis.

**Main text:**

Gut mucosal barriers are classified into chemical and physical barriers. Chemical barriers, including antimicrobial peptides (AMPs), are chemical agents that attack invading microorganisms, and physical barriers, including the mucus layer and the cell junction, are walls that physically repel invading microorganisms. These barriers, which are ingeniously modulated by gut microbiota and host immune cells, spatially segregate gut microbiota and the host immunity to avoid unnecessary immune responses to gut commensal microbes. Therefore, mucosal barrier dysfunction allows gut bacteria to invade gut mucosa, inducing excessive immune responses of the host immune cells, which result in intestinal inflammation.

**Conclusion:**

Gut mucosal barriers constructed by intestinal epithelial cells maintain gut homeostasis by segregating gut microbiota and host immune cells. Impaired mucosal barrier function contributes to the development of IBD. However, the mechanism by which the mucosal barrier is regulated by gut microbiota remains unclear. Thus, it should be further elucidated in the future to develop a novel therapeutic approach to IBD by targeting the mucosal barrier.

## Background

The mammalian intestine is a special place for microorganisms, where a high abundance of nutrients derived from foods are present and an aerobic condition is maintained. Therefore, tremendous numbers of microorganisms mainly composed of aerobic bacteria grow and inhabit the intestine. The intestinal microorganisms including bacteria, fungi and viruses form an ecological community termed the gut microbiota, which does not only reside in the gut but also provide many benefits to nutrition, metabolism and immunity. Short-chain fatty acid (SCFA), which is a gut microbial metabolite produced from dietary fibers, is used as an energy source of the host. In addition, SCFA contributes to the modulation of mucosal immunity by enhancing mucus production and promoting regulatory T cell (T_reg_) development [1–3]. Moreover, gut bacteria synthesize several kinds of vitamins including vitamin B and vitamin K, which are critical for sugar and fat metabolism and maintenance of hemostatic function. Thus, gut microbiota forms a win-win relationship with the host.

However, mammalian immune cells such as macrophages and neutrophils are programmed to attack invading extraneous organisms. Gut microbes are no exception and can be targeted by host immune cells. Accordingly, there is a barrier system—mucosal barrier—for separating gut microbiota and the host immunity to avoid an unfavorable interaction between the two. Mucosal barrier impairment allows gut microbes to easily enter the mucosa, which induce intestinal inflammation as a consequence of the host’s excessive immune responses to gut microbes.

Inflammatory bowel diseases (IBD) such as Crohn’s disease (CD) and ulcerative colitis (UC) involve choric intestinal inflammation in humans. Recent evidence based on the combination of the human genome-wide association study (GWAS) and genetically modified mouse studies have revealed that intestinal barrier dysfunction is one cause of IBD [4]. In addition, reduced production of mucosal barrier components such as mucus and antimicrobial peptides is observed in the intestine of some IBD patients. These findings indicate that the mucosal barrier is indispensable for maintaining the gut environment and preventing intestinal inflammation.

In this review, we discuss the mechanisms of the gut mucosal barrier constructed by IECs and the regulation of intestinal inflammation by the mucosal barrier.

### Mucosal barriers formed by intestinal epithelial cells

**Fig. 1:**
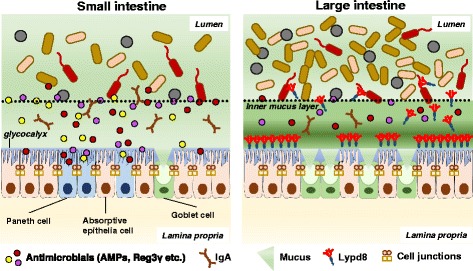
Mucosal barriers in the gut. Chemical barriers including AMPs and Reg3γ secreted by Paneth cells mainly contribute to the separation between intestinal bacteria and IECs in the small intestine. By contrast, in the large intestine where a tremendous number of bacteria exist, intestinal bacteria and IECs are largely segregated by physical barriers such as the inner mucus layer composed of polymerized MUC2 mucin. Lypd8, a highly glycosylated GPI-anchored protein expressed on IECs, inhibits the bacterial invasion of the inner mucus layer by binding to intestinal bacteria, especially flagellated bacteria. AMP: antimicrobial peptide

IECs at the surface of the gut mucosa absorb nutrients and water from ingested foods. They also play important roles in generating various types of barriers to protect mucosa from commensal microbes and invading pathogenic microorganisms (Fig. 1). These barriers have two subtypes, chemical and physical barriers.

### Chemical barrier

Chemical barriers consist of antimicrobial peptides (AMPs), the regenerating islet-derived 3 (Reg3) family of proteins, lysozyme and secretory phospholipase A2. All of these are mainly involved in the segregation of gut bacteria and IECs in the small intestine [5, 6]. Paneth cells play a crucial role in the mucosal barrier of the small intestine by producing a large number of antimicrobials [7].

AMPs are basic amino acid-rich cationic small proteins, which are evolutionally conserved in a wide range of organisms. They include the defensin family of proteins and cathelicidins, both of which bind to the negatively charged microbial cell membrane and induce disruption of membrane integrity by forming a pore-like structure [8]. Defensin family proteins are classified into α-, β- and θ-defensins, among which α-defensin (also referred to as cryptdins in mice) is most highly expressed in Paneth cells and mainly protects against infection by Gram-positive and Gram-negative bacteria. Pro-cryptdin is converted into mature-cryptdin by matrix metalloproteinase-7 (MMP-7) in mice. Therefore, MMP-7-deficient mice lack mature-cryptdin, resulting in high susceptibility to *Salmonella typhimurium* infection [9]. Moreover, mature α-defensin deficiency is associated with alteration of the gut microbiota: a decrease of Bacteroidetes and an increase in Firmicutes [10]. These results demonstrate that AMPs largely contribute to the homeostatic state of the gut environment by regulating pathogenic bacteria [11].

The Reg3 family proteins are C-type lectins, which exert an antibacterial effect on Gram-positive bacteria by binding to the bacterial membrane and forming a hexameric membrane-permeabilizing oligomeric pore [12]. In mice lacking Reg3γ, increased bacterial colonization on the epithelial surface of the small intestine was observed, indicating that Reg3γ is indispensable to the spatial separation of the intestinal bacteria and intestinal epithelia of the small intestine [6, 12, 13].

### Physical barriers

Chemical barriers are major players in the segregation of gut microbiota and the small intestinal epithelia. However, in the large intestine, where there is nothing resembling Paneth cells that secrete antimicrobials, physical barriers mainly contribute to spatial segregation of gut microbiota and intestinal epithelia. Physical barriers consist of the mucus layer covering the intestinal mucosa, the glycocalyx on the microvilli of absorptive IECs, and the cell junctions firmly linking IECs. These barriers physically inhibit the microbial invasion of the mucosa.

Mucus is a viscous fluid secreted by goblet cells. It is enriched in mucin glycoproteins that form large net-like polymers [14]. In the large intestine, where tremendous numbers of intestinal bacteria exist compared with the small intestine, the number of goblet cells is much higher and the large intestinal epithelia are covered by a thick two-layered mucus layer: the outer loose and the inner firm mucus layer [15]. These two mucus layers are constructed of goblet cell-secreted Mucin2 (MUC2) protein, which is a highly *O*-glycosylated protein, forming large net-like structures. The inner mucus layer is stratified and anchored to the intestinal epithelia, which does not allow gut bacteria to easily penetrate into the inner mucus layer and thereby keeps the inner mucus layer free of bacteria [15]. The inner mucus layer is converted into the outer mucus layer by the proteolytic processing of polymerized MUC2 by the host or gut bacteria. The outer mucus layer is inhabited by numerous bacteria, some of which use polysaccharides of MUC2 as an energy source; therefore, the absence of dietary fiber, a major energy source of intestinal bacteria, leads to the expansion of mucin-degrading species, resulting in the increase of inner mucus degradation [16].

Regarding the mechanism by which the inner mucus layer is free of gut bacteria, various antimicrobial molecules such as immunoglobulin A (IgA) and the defensin family of proteins transported or produced by IECs may be involved in protecting against bacterial invasion of the inner mucus layer [17]. Although higher numbers of bacteria exist in the large intestine, the expression level of antimicrobial molecules in the large intestine is not higher than that in the small intestine, indicating that there is another mechanism to inhibit gut microbial invasion of the large intestinal epithelia without killing bacteria.

Ly6/Plaur domain containing 8 (Lypd8) is a highly glycosylated GPI-anchored protein highly and selectively expressed on the mucosal surface of the large intestine. A recent study demonstrated that many intestinal bacteria, including *Escherichia* spp. and *Proteus* spp., invaded the inner mucus layer in Lypd8-deficient mice [18]. In addition, it was revealed that Lypd8 inhibited bacterial motility of flagellated bacteria such as *Escherichia coli* and *Proteus mirabilis* through binding to their flagella, thereby inhibiting their bacterial invasion of the colonic epithelia. These results indicate that Lypd8 contributes to the segregation of intestinal bacteria and the large intestinal epithelia [18].

As mentioned above, Muc2 and Lypd8 are highly glycosylated. Glycans of the physical barrier-related proteins are critical for maintaining their barrier function. In mice lacking the *O*-glycan core structure of the MUC2 protein, bacterial invasion of the colonic mucosa was observed [19]. With removal of *N*-glycans from Lypd8, the inhibitory effect of Lypd8 against bacterial attachment on Caco-2 cells was severely reduced [18]. Furthermore, mice devoid of Fut2, which mediates the transfer of fucoses to the terminal galactose on glycans in cell-surface glycoproteins, are highly susceptible to pathogenic bacteria infection [20, 21]. The glycocalyx, a meshwork of carbohydrate moieties of glycolipids or glycoproteins including transmembrane mucins, blocks bacterial invasion into the intestinal tissue as a second wall followed by the mucus layer. These findings indicate that glycans of barrier-related proteins generated by IECs are vital for physical barrier function.

For intestinal bacteria passing through the mucus layer and glycocalyx by evading various kinds of antimicrobial molecules from the host, cell junctions, including the tight and adhesion junctions linking epithelial cells, are the final wall to physically hamper the invasion into the intestinal tissue through the paracellular pathway. Hence, the perturbed gut integrity and permeability caused by disruption of the cell junction of IECs leads to microbial translocation, and the consequent leakage of bacteria or their metabolites into the gut tissue can induce a chronic or acute inflammatory response in the intestine [22, 23].

### Regulation of mucosal barrier function by gut microbiota and immune cells

Mucosal barrier function is regulated by various signals from gut microbiota and host immune cells. IECs express a variety of pattern recognition receptors, including Toll-like receptors (TLRs) and nucleotide-binding oligomerization domain-containing proteins (NODs) to directly sense bacterial components. The production of antimicrobial molecules by IECs is controlled by TLR4/MyD88 signaling and NOD2 signaling driven by gut microorganisms [5, 6, 24]. In mice deficient in NOD2 sensing muramyl dipeptides, which are conserved structures in bacterial peptidoglycans, the expression of defensins is substantially reduced, resulting in high susceptibility to *Listeria monocytogenes* infection [24]. Moreover, mice lacking MyD88 in IECs show the decreased production of AMPs, Reg3γ and mucus by IECs, and eventually they become highly susceptible to experimental colitis and enteric bacterial infection [25, 26]. In addition, recent studies demonstrated that NOD-like receptor family pyrin domain containing 6 (NLRP6), a member of the NOD-like receptor family of pattern recognition receptors, is necessary for mucus granule exocytosis from goblet cells [27].

Metabolites from gut bacteria also directly enhance the mucosal barrier function of IECs. Mucus secretion from goblet cells is upregulated by butyrate, one of the SCFAs provided by gut bacteria [28]. Recent evidence revealed that the expression of cell junction-associated molecules such as occludins and claudins in IECs is enhanced by indole, a metabolite of dietary tryptophan from commensal bacteria possessing tryptophanase, via Pregnane X receptor (PXR) stimulation [29, 30].

The mucosal barrier function of IECs is also enhanced by cytokines from immune cells activated by gut commensal bacteria or pathogenic bacteria. Segmented filamentous bacteria (SFB) is a type of commensal bacteria found in the mouse or rat intestine. The attachment of SFB to IECs strongly promotes Th17 cell differentiation in the lamina propria by inducing serum amyloid A (SAA) production by IECs [31, 32]. In addition, SFB facilitates type3 innate lymphoid cells (ILC3) to produce Interleukin (IL)-22 in an IL-23 receptor-dependent manner. In the case of *Citrobacter rodentium* infection associated with enteritis, a potent Th17 cell-mediated response is induced [32]. IL-17 and IL-22 produced by Th17 cells or ILC3 upregulate the secretion of AMPs and Reg3 family proteins by IECs, and induce the fucosylation of cell membrane proteins on IECs of the small intestine, which work to regulate commensal and pathogenic bacteria [20, 33]. When parasite infection occurs, tuft cells, taste-chemosensory epithelial cells, produce IL-25 which activates ILC2 to secrete IL-13. This induces Th2 responses, resulting in an enhancement of mucin production and goblet cell differentiation [34–36].

**Fig. 2:**
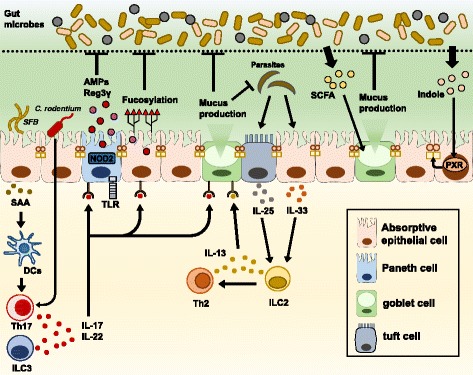
Regulation of mucosal barrier functions by gut microbes and host immune cells. Mucosal barrier function is modulated by gut microbes and host immune cells. SFB colonization or *C. rodentium* infection promotes the induction of helper T cells producing IL-17 and simulates ILC3 to secrete IL-22. Both cytokines enhance the production of antimicrobials such as AMPs and Reg3γ from IECs. In the case of parasite infection, activated tuft cells produce IL-25, which stimulates ILC2 to secrete IL-13. IL-13 promotes the proliferation of goblet cells and mucus production from them. Metabolites from gut microbes also directly influence the mucosal barrier function of IECs. SCFA promotes mucus production from goblet cells, and indole upregulates the expression of cell junction-related molecules through PXR activation SFB: segmented filamentous bacteria, SAA: serum amyloid A, ILC: innate lymphoid cell, TLR: Toll-like receptor, NOD2: nucleotide-binding oligomerization domain-containing 2, AMP: antimicrobial peptide, IEC: intestinal epithelial cell, SCFA: short-chain fatty acid, PXR: Pregnane X receptor.

In mucosal injury, IL-6 derived from intraepithelial lymphocytes enhances intestinal epithelial cell proliferation and contributes to healing from mucosal injury [37]. Moreover, activated macrophages differentiated from monocytes recruited to the mucosal wound site trigger the colonic epithelial progenitor niche with direct cell-cell contact to promote epithelial regeneration, which helps to recover the mucosal barrier [38]. Th2 cytokines, such as IL-5 and IL-13, promote colonic wound healing by inducing the alternative activation of macrophages, which contributes to epithelial cell proliferation [39]. Conversely, other pro-inflammatory cytokines, such as tumor necrosis factor (TNF)-α and interferon (IFN)-γ, inhibit epithelial cell proliferation through the suppression of β-catenin/T cell factor signaling [40]. Mucosal barrier function of IECs are maintained by intestinal microbiota and immune cell-derived cytokines (Fig. 2).

### Intestinal inflammation induced by the dysfunction of mucosal barriers

IBD is a group of chronic inflammatory states of the digestive tract, characterized by CD and UC. The incidence and prevalence of IBD are increasing around the world, suggesting that the elucidation of the pathogenesis of IBD is an emergent matter to be solved [41]. Recent remarkable advances of sequencing technology make it possible to identify various IBD susceptibility genes and the gut microbial composition of IBD patients. Accumulated evidence strongly indicates that both gut environmental factors including gut microbiota and host immune dysregulation associated with a genetic predisposition contribute to the occurrence and development of IBD [42]. IECs, which are present between gut microbiota and the host immunity, play an important role in the segregation of both factors by generating mucosal barriers to avoid excessive immune response to gut microbiota, which results in intestinal inflammation. Indeed, GWAS using next generation sequencing technology identified various IBD susceptibility genes including the mucosal barrier-related genes *FUT2*, *MUC19* and *NOD2* [43–46]. Additionally, the decreased production of mucosal barrier-related molecules, such as AMPs and mucins, is observed in the intestines of IBD patients [4].

To investigate the roles of mucosal barriers in preventing intestinal inflammation, many studies using genetically modified mice with mucosal barrier impairment have been conducted. Mice devoid of Muc2 show the disappearance of the inner mucus layer and develop spontaneous colitis resulting from the bacterial invasion of the colonic mucosa [15, 47]. The deficiency of cooperation of core 1 synthase (C1galt), which synthesizes the major constituent of the *O*-glycan core structure of the MUC2 protein, conduces to the disrupted mucus constitution and allows bacteria to invade the inner mucus layer, resulting in spontaneous colitis [19]. Abrogation of IEC fucosylation is associated with intestinal dysbiosis and leads to high susceptibility to intestinal inflammation. [48, 49] In mice deficient in Lypd8, a highly N-glycosylated protein expressed on IECs, the invasion of the colonic mucosa by a large number of flagellated bacteria such as *Proteus* spp. and *Escherichia* spp. causes high susceptibility to dextran sulfate sodium (DSS)-induced intestinal inflammation [18]. The absence of NLRP6 in IECs impairs mucus secretion from goblet cells, consequently leading to the disappearance of the bacteria-free zone just above the colonic epithelia. This is accompanied with high sensitivity to DSS-induced or bacterial pathogen-induced colitis [27, 50]. Interestingly, wild-type mice cohoused with NLRP6-deficient mice show high susceptibility to DSS-induced intestinal inflammation, indicating colitogenic dysbiosis of NLRP6-deficient mice is transmissible to normal mice [50]. The dysfunction of cell junctions also causes intestinal inflammation. Intestinal deletion of Claudin-7, which is a critical component of the tight junctions of IECs, enhances the paracellular flux of a bacterial product and consequently causes spontaneous colitis in mice [23]. In addition, in the absence of RING finger protein (RNF) 186, which acts as an E3 ligase to mediate polyubiquitination of its substrates, the sensitivity to intestinal inflammation is elevated because of the high permeability of small organic molecule and enhanced endoplasmic reticulum (ER) stress in IECs [51].

The impairment of chemical barriers also causes high susceptibility to intestinal inflammation. Mice devoid of IL-22 which enhances the production of antimicrobials by IECs also show high sensitivity to DSS colitis, indicating IL-22 from T cells is protective against intestinal inflammation [52]. Moreover, intestinal epithelial cell-specific inhibition of nuclear factor (NF)-κB through the conditional ablation of NEMO, an IκB kinase subunit essential for NF-κB activation, causes chronic intestinal inflammation in mice because of bacterial translocation into the colonic mucosa due to the reduced production of antimicrobial peptides [53]. Mice deficient in the *Nod2* gene, which is a susceptibility gene for human CD, do not show spontaneous intestinal inflammation but show severe Th1-driven granulomatous inflammation of the ileum induced by *Helicobacter hepaticus* because of the decreased expression of AMPs by Paneth cells [54–56]. The deficiency of multi-drug resistance protein 1 (MDR1), a xenobiotic transporter, leads to chronic colitis because of the increased permeability of IECs [57]. Deficiency in adaptor protein (AP)-1B, which mediates the sorting of membrane proteins, induced the reduced expression of antimicrobial proteins and the impaired secretion of IgA, leading to chronic colitis with an enhanced Th17 response [58].

**Fig. 3:**
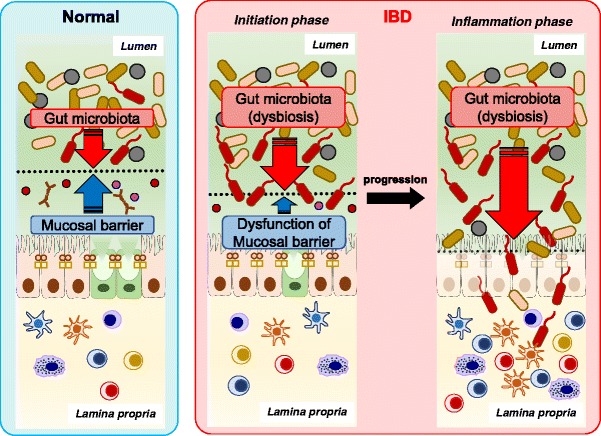
The imbalance between mucosal barriers and gut microbes promotes susceptibility to intestinal inflammation. In the steady state, intestinal bacteria and mucosal barriers maintain a well-balanced relationship, and thus intestinal bacteria and IECs are clearly segregated in the gut. However, dysfunction of mucosal barriers including decreased production of mucin or AMPs due to genetic factors and dysbiosis induced by environmental factors such as high-fat diet or various antibiotics disrupt the well-balanced relationship, and thereby intestinal bacteria can gain access to the gut immune cells, leading to the progression of IBD. IBD: inflammatory bowel disease

As described above, many human and mouse studies have demonstrated that intestinal barrier dysfunction is clearly implicated in the development of intestinal inflammation, indicating that the segregation of gut microbiota and host immunity by the mucosal barriers is critically involved in maintaining gut homeostasis (Fig. 3).

## Conclusions

IECs generate various kinds of mucosal barriers to segregate gut microbiota and gut immune cells to prevent excessive immune responses leading to intestinal inflammation. Accordingly, a defect in mucosal barrier function promotes the development of intestinal inflammation such as IBD. There are three major players involved in the pathogenesis of IBD. These include gut microbes in the lumen, immune cells in the lamina propria and IECs between the two. Regarding therapies for IBD, there are several immunosuppressive agents such as mesalazine, steroids and infliximab. Recently, fecal transplantation has been developed to improve the gut environment. However, extremely few therapies targeting the mucosal barrier function of IECs exist. The therapies for intractable IBD are limited, and several different immunosuppressive therapies are required, each having at least a few side effects. Further clarification of the mechanisms regulating the gut mucosal barrier system will certainly shed light on the development of novel therapeutic approaches for IBD.

## Abbreviations

AMP: Antimicrobial peptide
AP: Adaptor protein
C1galt: Cooperation of core 1 synthase
CD: Crohn’s disease
DSS: Dextran sulfate sodium
ER: Endoplasmic reticulum
GWAS: Genome-wide association study
IBD: Inflammatory bowel disease
IEC: Intestinal epithelial cell
IFN: Interferon
IgA: Immunoglobulin A
IL: Interleukin
ILC: Innate lymphoid cell
Lypd8: Ly6/Plaur domain containing 8
MDR: Multi-drug resistance protein
MMP-7: Matrix metalloproteinase-7
NEMO: Inhibitor of nuclear factor kappa B kinase subunit gamma
NF: Nuclear factor
NLRP6: NOD-like receptor family pyrin domain containing 6
NOD2: Nucleotide-binding oligomerization domain-containing protein 2
PXR: Pregnane X receptor
Reg3: Regenerating islet-derived 3
RNF: RING finger protein
SAA: Serum amyloid A
SCFA: Short-chain fatty acid
SFB: Segmented filamentous bacteria
TLR: Toll-like receptor
TNF: Tumor necrosis factor
T_reg_: Regulatory T cell
UC: Ulcerative colitis
